# Pupylation-Based Proximity-Tagging of FERONIA-Interacting Proteins in Arabidopsis

**DOI:** 10.1016/j.mcpro.2024.100828

**Published:** 2024-08-13

**Authors:** Zhuoran Lin, Di Liu, Yifan Xu, Mengyang Wang, YongQi Yu, Andrew C. Diener, Kun-Hsiang Liu

**Affiliations:** 1 State Key Laboratory of Crop Stress Biology for Arid Areas and College of Life Sciences, Northwest Agriculture & Forestry University, Yangling, Shaanxi, China; 2 Department of Molecular Biology, Centre for Computational and Integrative Biology, Massachusetts General Hospital, Boston, Massachusetts, USA; 3 Department of Genetics, Harvard Medical School, Boston, Massachusetts, USA; 4 Institute of Future Agriculture, Northwest Agriculture & Forestry University, Yangling, Shaanxi, China

**Keywords:** Arabidopsis, receptor-like kinase, FERONIA, interactome, pupylation-based proximity-tagging, LC-MS/MS, leaf, seedling, flower

## Abstract

The plasma membrane-localized receptor kinase FERONIA (FER) plays critical roles in a remarkable variety of biological processes throughout the life cycle of *Arabidopsis thaliana*. Revealing the molecular connections of FER that underlie these processes starts with identifying the proteins that interact with FER. We applied pupylation-based interaction tagging (PUP-IT) to survey cellular proteins in proximity to FER, encompassing weak and transient interactions that can be difficult to capture for membrane proteins. We reproducibly identified 581, 115, and 736 specific FER-interacting protein candidates in protoplasts, seedlings, and flowers, respectively. We also confirmed 14 previously characterized FER-interacting proteins. Protoplast transient gene expression expedited the testing of new gene constructs for PUP-IT analyses and the validation of candidate proteins. We verified the proximity labeling of five selected candidates that were not previously characterized as FER-interacting proteins. The PUP-IT method could be a valuable tool to survey and validate protein-protein interactions for targets of interest in diverse subcellular compartments in plants.

## Highlights

• PUP-IT was used to survey proteins near the plasma membrane receptor FERONIA (FER).
• PUP-IT found FER-interacting proteins in protoplasts, seedlings, and flowers.
• The study identified five new FER-interacting proteins, expanding the FER interactome.

## Graphical abstract

**Figure:**
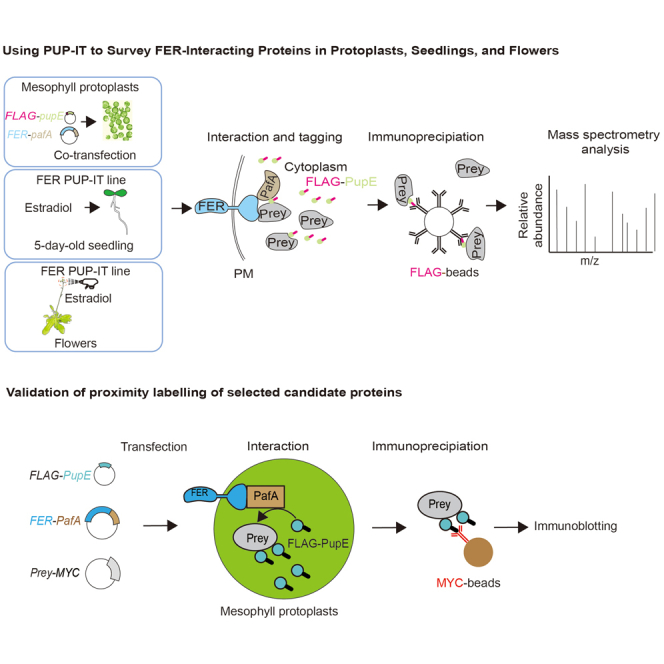

## In Brief

FERONIA (FER), a plasma membrane-localized receptor kinase in *Arabidopsis thaliana*, is crucial in various biological processes. Employing pupylation-based interaction tagging (PUP-IT), we identified 581, 115, and 736 specific FER-interacting proteins in protoplasts, seedlings, and flowers. Fourteen known FER-interacting proteins were validated, and protoplast transient gene expression expedited the testing of new gene constructs. This study highlights the versatility of PUP-IT in surveying and validating protein-protein interactions, offering insights into FER's role in plants.

Molecular characterization of a cellular process typically involves breaking the process up into a series of protein-protein interactions having specific functions. The consequences of protein interactions, which may be transient or persistent, determine the localization, organization, modification, activity, and stability of those proteins (1). Choosing the most appropriate method to detect particular protein–protein interactions or discover novel interactions depends on factors such as the duration and nature of the interactions and whether the interactions modify the proteins; moreover, not all methods are amenable to high-throughput and discovery of novel interactions (1, 2, 3).

Proximity-labeling methods are potentially valuable in broad applications of biological research for their versatility and sensitivity (4, 5, 6, 7, 8, 9, 10). A bait protein fused with a specific enzyme (mostly engineered ligase or peroxidase) directs the labeling of prey proteins that come into its proximity. Because labeling is a rapid covalent chemical modification resulting in a stable tag on prey proteins, even prey proteins with weak, transient, or no direct contact may be captured by tagging. The resulting tag provides a means to enrich and detect specific prey proteins (10). However, each proximity-labeling method has challenges and limitations.

In the most prevalent proximity-labeling methods, the bait protein is genetically fused to a promiscuous variant of biotin ligase BirA of *Escherichia coli* (11). The variant biotin ligase constitutively produces a reactive intermediate of biotin, which diffuses and reacts with primary amines on prey proteins in proximity to the bait-biotin ligase fusion (12, 13, 14, 15). Proximity-labeling occurs when an excess of exogenous biotin is applied to cells because the variant biotin ligase needs sufficient intracellular biotin to appreciably generate reactive intermediates (9, 10). As plants synthesize biotin, controlling activity by biotin depletion is relatively challenging. The abundance of endogenous peroxidase rendered it prohibitive for application of the proximity-labeling method based on monomeric peroxidase APEX2 in plants (10, 16).

In an alternative new proximity-labeling method, the bait protein is genetically fused to PafA ligase of *Corynebacterium glutamicum* (5, 17). Performing a role analogous to eukaryotic ubiquitin ligases, PafA normally ligates the deaminated carboxy-terminal Glu of the 64-amino acid PupE to the side-chain amine of lysines on target proteins destined for degradation in a process termed pupylation in bacteria. For applications in eukaryotes by fusing PafA to the bait, ligation of PupE is instead directed to prey proteins in proximity or interacting with the bait. The amino-terminal end of PupE can be modified with a variety of epitopes or affinity tags. Gene fusions for both *PafA* and *pupE* are co-expressed in cells to perform proximity labeling analyses (5, 18).

Regarding proximity labeling, the pupylation of PafA differs from the biotinylation of BirA in at least three ways (5, 9, 10, 18). For one, activated PupE remains bound to PafA until reaction with prey while activated biotin diffuses away from BirA. This makes the labeling radius of Pup more restrictive. On the other hand, the much larger size of the Pup may diminish access to prey in comparison to biotin. For two, PupE is co-expressed with the bait-PafA fusion protein while biotin is exogenously supplied to cells that do not synthesize or accumulate biotin. In some tissues and organs, the inaccessibility of cells and poor uptake of biotin may complicate biotinylation. For three, PupE does not exist in eukaryotes while biotin is an essential cofactor and natural covalent attachment to some enzymes. Thus, unlike biotin, no inherent background is present to interfere with the detection or enrichment of PupE.

FERONIA (AT3G51550) is one of hundreds of plasma membrane-spanning receptor-like kinases (RLKs) in Arabidopsis and one of 17 members of the *Catharanthus roseus* RLK-1-like kinase (CrRLK1L) subfamily and has garnered outsized attention among its homologs because of the pleiotropy of its gene mutations (19, 20). The *fer* mutants exhibit defects throughout the lifecycle of Arabidopsis, including aberrant growth and development of both the root and shoot, delay in flowering time, and female semi-sterility (20, 21). Moreover, *fer* plants exhibit defects in responses to hormones, various biotic and abiotic stressors, and the availability of nutrients. Arguably this pleiotropy may partially be a consequence of a common underlying role of *FER* in the homeostasis of cell turgor and cell wall integrity (20, 21, 22, 23, 24).

Matching the remarkable pleiotropy of *fer*, multiple extracellular signals, such as the secreted peptide family of rapid alkalinization factors, leucine-rich repeat extension proteins, and de-esterified pectin, are transduced by FER into a variety of intracellular responses including calcium signaling, generation of reactive oxygen species, nitric oxide and ethylene, modulation of hormone signaling, alkalinization of the apoplast and transcriptional reprogramming. The consequences of numerous protein interactions in specific responses are described in reviews of FER (20, 21, 25). Nevertheless, the catalog of FER-interacting proteins undoubtedly remains incomplete.

We have used the pupylation-based interaction tagging (PUP-IT) method to show the specific interaction of the TOR complex and FIE subunit of the PRC2 complex in Arabidopsis mesophyll protoplasts (18, 26). Here we extended the applications of PUP-IT and made surveys of Arabidopsis proteins that came into proximity to FER in mesophyll protoplasts, seedlings, and flowers. We identified candidate FER-interacting proteins in the analysis of proteins that were enriched for FER-directed pupylation using liquid chromatography and tandem mass spectrometry (LC-MS/MS). While some identified candidates were previously characterized as FER-interacting proteins, most were not. With a selection of five novel candidates, we used PUP-IT analyses to validate their proximity to FER.

## Experimental Procedures

### Experimental Design and Statistical Rationale

The main objective of the study was to investigate the potential interacting proteins of the receptor-like protein kinase FERONIA. Mass spectra of fragmented proteins were obtained from lysates of *Arabidopsis thaliana* mesophyll protoplasts and seedlings or flowers of the PUP-IT FER transgenic line. There were three repeats for samples of the experimental group and three repeats for samples of the negative control. All mass spectra were analyzed using Proteome Discover (version 2.2). The false discovery rate at the protein level is 0.01 (strict) and 0.05 (relaxed). Altogether, the dataset, including raw data files and search results, was uploaded to PRIDE (see below). For the volcano plot analysis, quantitative data derived from the analysis of mass spectra were filtered. Significant enrichment of proteins was determined using Perseus (2.0.11) for analysis of the output file of Proteome Discover (version 2.2). Abundances (Normalized) values were used for analysis. Razor Peptides of proteins number more than one was filtered out. The normalized abundance values were transformed using a log_2_(x) base. The proteins were further filtered, removing proteins that had normalized abundances values from fewer than three samples (out of the six experimental and control samples). Missing values were replaced by imputation, using a normal distribution with a width of 0.3 and down shift of 1.8 and selecting the total matrix option. Filtered data were visualized in volcano plots. Volcano plots and the principal component analysis (PCA) test (shown in Supplemental Fig. S1) were performed using ggplot2 and factoextra of R (version 4.3.3). The R script is provided in Supplemental Table 1. All statistical tests were two-sided, and statistical significance was defined as a *p*-value <0.05.

### Plasmid Constructs and Transgenic Lines

Oligonucleotide primers used to PCR-amplify DNA fragments are listed in Supplemental Table 2. DNA fragments *pupE, pafA, 6xHis-3xFLAG-pupE, and pafA-GS-linker* were commercially synthesized (Tsingke, see Supplemental Fig. S2 for DNA sequences). To construct plasmid *HBT-6xHis-3xFLAG-pupE-NOS, 6xHis-3xFLAG-pupE* was PCR-amplified and subcloned into vector *HBT-NOS* linearized by restriction digestion with *Bam*HI and *Pst*I (27). To construct the binary plasmid *FER-PUP-IT*, the three DNA fragments *6xHis-3xFLAG-pupE-NOS, pFER-FER-GS-linker-pafA*, and *HA-NOS* were PCR-amplified and subcloned, using Gibson assembly (Vazyme, C112-01), into the vector *pER8* linearized by restriction digestion with *Xho*I and *Spe*I (28). The template DNA for the PCR of *6xHis-FLAG-pupE-NOS, pFER-FER-GS-linker-pafA* and *HA-NOS* were the plasmid *HBT-6xHis-3xFLAG-pupE-NOS*, Arabidopsis genomic DNA, and plasmid *HBT-HA-NOS*, respectively. Using the floral-dip method, Agrobacterium strain GV3101 harbouring *FER-PUP-IT* mediated transformation of Arabidopsis plants (29). The resulting T_1_ seeds were selected for hygromycin B resistance (50 μg/ml) on agar plates containing 0.5× MS basal salts. To construct plasmid *HBT-6xHis-3xFLAG-pupE-NOS*, the DNA fragment *6xHis-3xFLAG-pupE* was PCR-amplified from plasmid *FER-PUP-IT* and subcloned into plasmid the *HBT-FLAG-NOS* linearized by restriction digestion with *Bam*HI and *Stu*I (27). To construct plasmid *FER-pafA*, the DNA fragment *FER-GS-linker-pafA* was PCR-amplified from plasmid *FER-PUP-IT* and subcloned into the plasmid *HBT-HA-NOS* plasmid linearized by restriction digestion with *Bam*HI and *Stu*I. To construct plasmid *PUP-IT-NEG*, three DNA fragments *6xHis-3xFLAG-pupE-NOS, UBQ10-pafA-GS linker* and *HA-NOS* were PCR-amplified and combined using Gibson assembly (Vazyme, C112-01) with vector *pCAMBIA1300* linearized by restriction digestion with the *Bam*HI and *Xba*I. The DNA fragment *6xHis-3xFLAG-pupE-NOS* was PCR-amplified from the plasmid *pER8-FER-PUP-IT*, and the DNA fragment *HA-NOS* was PCR-amplified from the plasmid *HBT-HA-NOS*. To generate DNA fragments *UBQ10-pafA-GS* linker, two PCR products, *UBQ10* and *pafA-GS* linker (having sequence overlap respectively at the 3′ and 5′ ends), were combined using overlap extension PCR; *UBQ10* and *pafA-GS*-linker were PCR-amplified respectively from plasmids *UBQ10-GUS-NOS* and *pER8-FER-PUP-IT*. To construct plasmid *pFER-FER-GFP-HA* and *pFER-FER-pafA-GFP-HA*, the *FER* native promoter, *FER* coding region, and *FER-pafA* fragment were PCR-amplified from the plasmid *pER8-FER-PUP-IT* and combined using Gibson assembly (Vazyme, China, C112-01) with vector *pCAMBIA1300* linearized by restriction digestion with the *EcoR*I and *Stu*I (30). To construct plasmids *UBQ10-GRF3-MYC-NOS, UBQ10-TSM1-MYC-NOS, UBQ10-AGD2-MYC-NOS, UBQ10-MSL10-MYC-NOS*, and *UBQ10-CKX2-MYC-NOS*, coding sequences of *GRF3*, *TSM1*, *CKX2*, *AGD2*, and *MSL10* were PCR-amplified from Arabidopsis cDNA and subcloned into plasmid *UBQ-MYC-NOS* linearize by restriction digestion with *Kpn*I and *Stu*I (31).

### Plant Materials and Growth Conditions

Arabidopsis ecotype Columbia (Col-0) was used throughout. Arabidopsis mesophyll protoplasts were obtained from seedlings grown at 23 °C in 12 h light for 25 days. For PUP-IT analyses in seedlings, 300 seeds of the transgenic line were sown per dish (100 mm × 10 mm) in 10 ml of 1× MS basal salts (PhytoTech, M519), 0.1% MES, 1% sucrose, at pH 5.8 in constant light (60 μmol m^−2^s^−1^). After 5 days, 10 μl 10 μM estradiol (dissolved in DMSO) or DMSO was added to medium, and seedlings were left in liquid culture for two more days. Seedlings from two dishes were pooled for one sample. For PUP-IT analyses in flowers, the transgenic line was grown on soil for 35 days with a daylength of 16 h under fluorescent lighting (100 μmolm^−2^s^−1^) at 23°C (day)/21°C (night). Plants were sprayed twice daily with 10 μM estradiol/0.1% DMSO or 0.1% DMSO (control) for 3 days.

### Mesophyll Protoplasts Transient Expression Assays

Arabidopsis mesophyll protoplasts were prepared and transfected as previously described (27). For PUP-IT analyses with FER-PafA, 10^6^ protoplasts in 5 ml were mixed with plasmid DNA, 350 μg of *HBT-FER-pafA-HA*, and 150 μg of *HBT-FLAG-pupE* for co-transfection. For PUP-IT analyses with PafA alone, 500 μg of the plasmid *PUP-IT* was mixed with protoplasts instead. Protoplasts were incubated in 25 ml WI buffer for 12 h. To validate PUP-IT analyses of candidate proteins, 10^6^ protoplasts were mixed with plasmids 300 μg of *HBT-FER-pafA-HA* plasmid, 150 μg of *HBT-FLAG-pupE*, and 300 μg of *UBQ-Prey-MYC (UBQ-GRF3-MYC, UBQ-TSM1-MYC, UBQ-AGD2-MYC, UBQ-CKX2-MYC, UBQ-MSL10-MYC*, or *UBQ-GFP-MYC)* for co-transfection. Protoplasts were then incubated in WI buffer for 12 h.

### FER and FER-PafA Subcellular Localization Assay

To determine the subcellular localization of FER-GFP and FER-PafA-GFP, 2 × 10^4^ protoplasts were mixed with 20 μg of *pFER-FER-GFP-HA* or *pFER-FER-pafA-GFP-HA* plasmid and incubated in 1 ml of WI buffer for 12 h. The protoplasts were loaded onto slides and imaged with the 20×objective lens on a laser scanning confocal microscopy (LSCM, Leica Stellaris 8). To detect expression of FER-GFP-HA and FER-pafA-GFP-HA protein, lysates of 100 μl cells were separate on SDS-PAGE and transferred onto a Western blot for immunodetection using anti-HA antibody (Abmart, M2003L, 1:5000).

### Immunoprecipitation

For PUP-IT analyses in mesophyll protoplasts, cells were lysed in 100 μl of extraction buffer, consisting of 150 mM NaCl, 50 mM Tris-HCl pH 7.5, 5 mM EDTA pH 8.0, 1% Triton X-100, 1 mM DTT and 1× proteinase inhibitor cocktail (MedChemExpress, HY-K0011), supplemented with 1% SDS. Sample was lysed on the ice for 5 min, and then 1 ml extraction buffer was added to dilute SDS in the lysate. The protein extract was centrifuged at 1,000g for 10 min at 4 °C, and FLAG-conjugated protein in the supernatant was immunoprecipitated with 25 μl anti-FLAG magnetic beads (BioMag, BMFA-300-2-1). For PUP-IT analyses in seedlings and flowers, tissue was ground in liquid nitrogen to a powder and resuspended and lysed in 300 μl extraction buffer supplemented with 1% SDS on ice for 5 min, and then diluted with 3 ml extraction buffer for another 5 min. The protein extract was centrifuged at 1,000*g* for 10 min at 4 °C, and FLAG-conjugated protein in the supernatant was immunoprecipitated with 35 μl anti-FLAG beads.

### Immunoblot Analyses

To confirm the expression of FER-PafA-HA and FLAG-tagged proteins in PUP-IT experiments, protein extract (20 μl) of protoplasts or protein extract (40 μl) of seedling and flower samples were subjected to SDS–PAGE and immunoblotting with anti-FLAG (Abmart, China, M2008M, 1:5000) or anti-HA (Abmart, M2003L, 1:5000) antibodies. For validation of candidate FER-Interacting proteins, tagged proteins were immunoprecipitated with 30 μl anti-MYC beads (MedChemExpress, HY-K0206) and then eluted with 1× Laemmli sample buffer (100 mM Tris-HCl pH 6.8, 200 mM DTT, 4% SDS, 0.2% bromophenol blue, 20% glycerol).

The proteins eluted from the beads were size-separated by SDS-PAGE and blotted onto PVDF membranes by semi-dry transfer. Tagged proteins on membranes were detected with anti-FLAG (Abmart, M2008M, 1:5000) or anti-HA (Abmart, M2003L, 1:5000), or anti-MYC (Abmart, M2002M, 1:5000) antibodies. To detect the expression of FER-GFP or FER-PafA-GFP protein, 10^4^ transfected protoplasts described above were lysis with 1× Laemmli sample buffer and subjected to SDS-PAGE and immunoblotting with α-HA antibody (Abmart, China, M2003L, 1:5000).

### Protein Digestion and Peptides Enrichment

Protein immunoprecipitated on anti-FLAG beads was eluted with 20 μl of 2× Laemmli sample buffer and separated by SDS-PAGE. Gel slices containing tagged proteins were subjected to in-gel digestion with Trysin (Thermo fisher scientific, 90059), and resulting peptides were purified and enriched using Thermo fisher scientific Pierce C18 Tips (Thermo fisher scientific, 87782).

### Liquid Chromatography Tandem Mass Spectrometry

Purified enriched peptides were dissolved in 10 μl of 0.1% formic acid (FA) in water and then injected into an Easy-nLC 1200 system (Thermo Fisher Scientific). They were separated on a 15-cm Easy-Spray column (75 μm ID) containing C18 resin (5 μm). The mobile phase buffer consisted of 0.1% FA in ultra-pure water (Buffer A), and the eluting buffer was 0.1% FA in 80% acetonitrile (ACN, Buffer B), running over a linear 42-min gradient from 12% to 40% of Buffer B at a flow rate of 300 nl/min. The Easy-nLC 1200 was coupled online with an Orbitrap Fusion Lumos mass spectrometer (Thermo Fisher Scientific).

For protein assignment of peptides, the mass spectrometer operated in data-dependent acquisition (DDA) mode and performed a full MS scan from m/z 375 to 1500 with a resolution of 60,000 at m/z 200. The full MS AGC (Automatic Gain Control) value was set to 4 × 10^5^ with a maximum injection time (IT) of 100 ms. Normalized collision energy (NCE) was set at 32%. The AGC for the fragment spectra was 5 × 10^4^ with an IT of 512 ms. The isolation width was set at 1.6 m/z, and dynamic exclusion was set to 60 s. MS2 spectra were converted to peak list files, and these files were searched using Proteome Discover (version 2.2) against a TAIR10 database (40,746 entries, downloaded on 12/07/2021) from TAIR concatenated with sequence-randomized versions of each protein. The searching parameters were set as follows: digestion enzyme - trypsin (full), maximal missed cleavage - 2, minimal peptide length - 6, precursor mass tolerance - 10 ppm, static modifications - cysteine residues carbamidomethylation (+57.021 Da), dynamic modifications included N-terminus acetylation (+42.011 Da), methionine residues oxidation (+15.995 Da). For label free quantitation, the general quantification settings in Proteome Discover (version 2.2) were set as follows: “peptides to use” was “Unique + Razor,” “consider protein groups for peptide uniqueness” was “True, “reject quan results with missing channels” was “False,” “precursor abundance based on” was “Intensity,” “normalization mode” was “total peptide amount” and “scaling mode” was “on all average.” The false discovery rate at the protein level is 0.01 (strict) and 0.05 (relaxed). The first peptide precursor mass tolerance was set at 10 ppm, and the MS/MS match tolerance was set at 20 ppm.

### Bioinformatics Analysis

Gene annotation was generated from the website (https://bar.utoronto.ca/ntools/cgi-bin/ntools_classification_superviewer.cgi).

## Results

### Surveying Candidate Proteins for Proximity to FER in Arabidopsis Protoplasts

**Figure 1:**
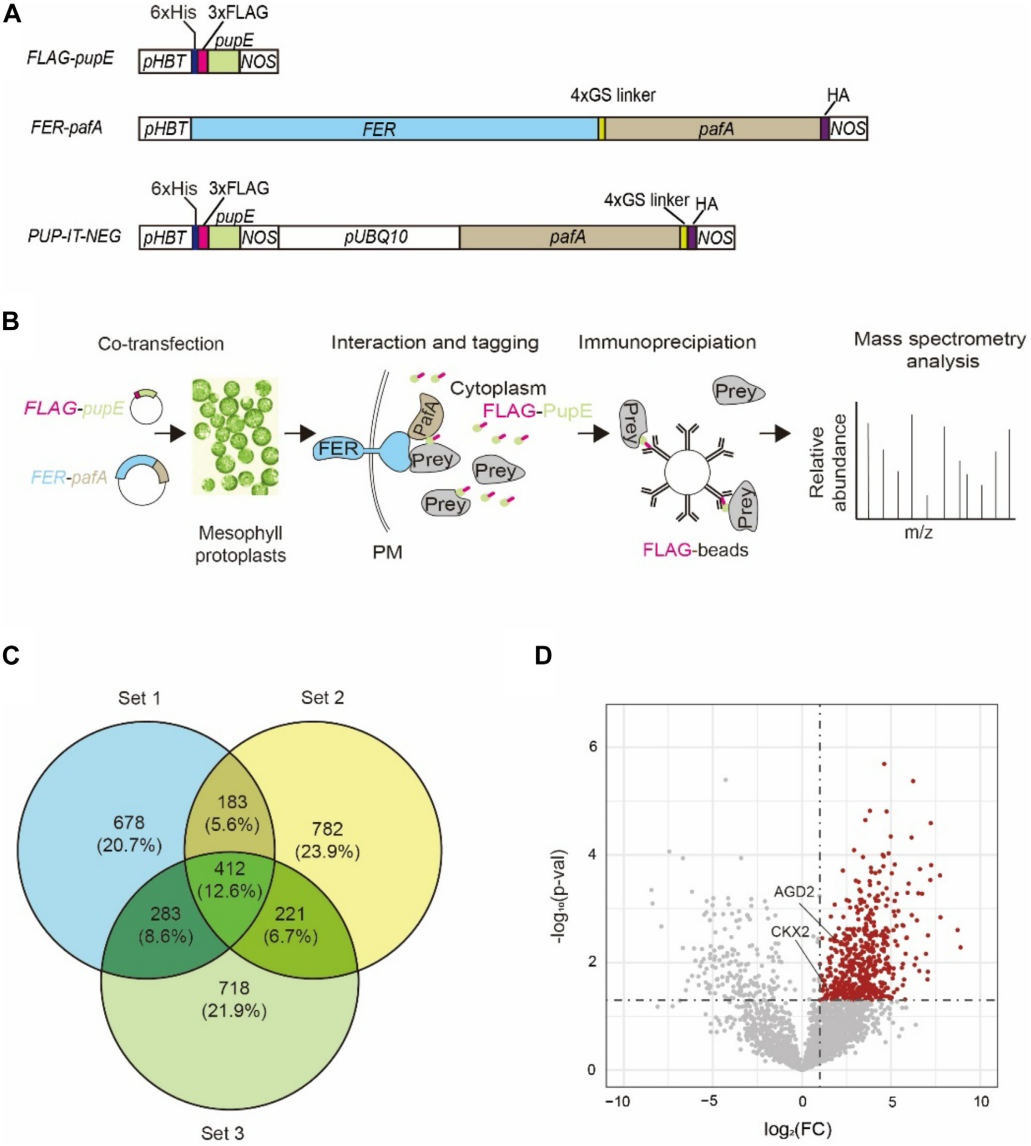
**Identifying FER-proximal proteins by PUP-IT analyses in Arabidopsis protoplasts.***A*, Gene constructs for transient expression in mesophyll protoplasts. Transcriptional promoter (*pHBT*) and termination (*NOS*) sequences respectively flank start and stop codons. *FLAG-pupE* includes coding sequence for six consecutive histidines (6xHis), three consecutive FLAG epitopes (FLAG) and the activated intermediate of *Corynebacterium glutamicum* Pup (*pupE*). *FER-pafA* includes coding sequence for Arabidopsis FER (*FER*), four repeats of the amino acid sequence Gly-Gly-Gly-Ser (GS linker), *C. glutamicum* PafA ligase (*pafA*) and two consecutive HA epitopes (HA). The *PUP-IT-NEG* construct includes two genes, the first of which is *FLAG-pupE*. The second is flanked by the promoter of Arabidopsis *UBQ10* (*pUBQ10*) and *NOS* and includes coding sequence of *pafA*, GS linker and HA. *B*, Schematic diagram of PUP-IT for FER-interaction tagging in Arabidopsis protoplasts. Transfection introduced *FLAG-pupE* and *FER-PafA* to mesophyll protoplasts. Interaction of prey proteins and plasma membrane (PM)-localized FER-PafA led to ligation of FLAG-PupE to prey proteins. Immunoprecipitation with anti-FLAG antibody-beads enriched FLAG-tagged prey proteins. Analysis of peptides from prey proteins using mass spectrometry identified candidate proteins. *C*, overlap of three sets of candidate proteins from three replicated experiments is depicted in a Venn diagram with numbers and percentages of proteins. *D*, Volcano plot of the 2464 (*red and grey*) proteins identified by LC–MS/MS in protoplasts. The −log_10_ (*p*-value) is plotted against the log_2_ (fold change: abundant proteins in the experiment group/abundant proteins in the control group). The non-axial vertical lines denote ±1.0-fold change while the non-axial horizontal line denotes *p* = 0.05. The *red dots* represent 491 proteins with significant differences between the experimental group and the control (*p*-value <0.05 and fold change >1).

We first deployed PUP-IT analyses in Arabidopsis mesophyll protoplasts. A comparative advantage of using protoplasts rather than whole plants is the high efficiency and speed (hours *versus* months) of testing newly created gene constructs. To target pupylation to cytosolic proteins that come into proximity to FER, we generated a gene fusion (*FER-pafA*) of coding sequences of *FER* and *pafA* that could be transiently expressed in protoplasts (Fig. 1*A*). In the resulting fusion protein, the carboxy-terminus of FER, which would reside on the cytoplasmic side of the plasma membrane, is extended with a repeated linker sequence of Gly-Gly-Gly-Ser and followed by the Pup ligase PafA (32). Similar to FER-GFP protein, the FER-PafA-GFP fusion protein localized on the plasma-membrane (Supplemental Fig. S3) (22, 33). To enable detection of pupylated protein by FER-PafA, we generated a fusion of sequence encoding three copies of FLAG epitopes to the coding sequence of PupE as activated PafA substrate (FLAG-PupE) for co-expression in plant protoplasts (Fig. 1*A*).

Transient co-expression of FER-PafA and PupE-FLAG in transfected protoplasts was expected to comprehensively detect FLAG-epitope tag cytosolic proteins that came into proximity to FER (Supplemental Fig. S4*A*). Protoplasts were lysed 12 h after transfection, and FLAG-tagged proteins were immunoprecitated with anti-FLAG antibody immobilized on magnetic beads. The identities of peptides derived from trypsin digestion of isolated proteins were subsequently predicted using LC-MS/MS (Fig. 1*B*). In triplicated experiments, sufficient proteins were obtained to identify peptides of 3317 Arabidopsis proteins in total (Fig. 1*C* and Supplemental Table S3). Among the three sets of identified proteins, 412 were present in all three replicates, and 687 were in at least two sets. The numbers and percentages of identified proteins among the three sets including their overlap are shown in Figure 1*C*. GLYCINE-RICH RNA-BINDING PROTEIN 7 (GRP7, At2g21660), PLANT U-BOX 9 (PUB9, At3g07360), and ARABIDOPSIS H(+)-ATPASE 2 (AHA2, At4g30190), which are characterized FER-interacting proteins, were among candidate proteins in two of three replicates (34, 35, 36).

Associations of cytosolic proteins with PafA alone might be responsible for their proximity to FER-PafA. To take into account the pupylation and FLAG-tagging resulting from proximity to PafA, we conducted a control experiment in which *pafA*, in place of *FER-pafA*, was co-expressed with *FLAG-pupE* in transfected protoplasts. The same experimental and analytical procedures were used to isolate and identify FLAG-tagged proteins as above (Supplemental Table S4). Predicted peptides could identify 5964 Arabidopsis proteins in replicated experiments (Supplemental Table S4). Among the proteins pupylated by FER-PafA, 491 were determined to be significantly pupylated by PafA under the conditions of a *p*-value <0.05 and a fold change >1 cutoff (Fig. 1*D*, and Supplemental Tables S5 and S6).

### Surveying FER-Interacting Proteins in Arabidopsis Seedlings

**Figure 2:**
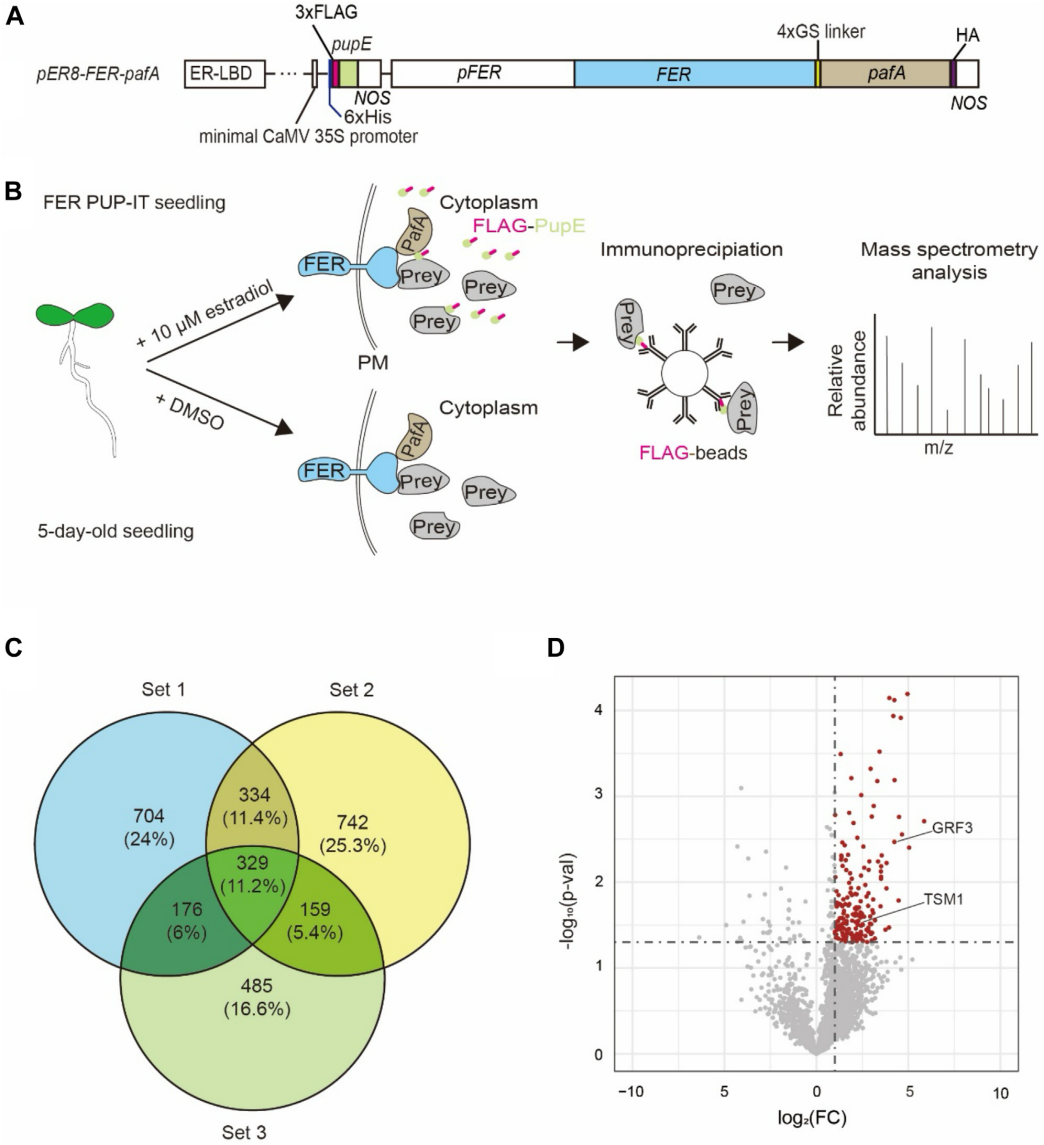
**Identifying FER-proximal proteins by PUP-IT analyses in Arabidopsis seedlings.***A*, transfer DNA (T-DNA) region of the binary plasmid used for Agrobacterium-mediated transformation of Arabidopsis includes three genes *XVE*, *8xLexO*-*FLAG-pupE* and p*FER*-*FER-pafA*. *XVE* constitutively expresses an estrogen receptor-based transactivator (27). The promoter sequence *8xLexO* makes transcriptional expression of *FLAG-pupE* (described in Fig. 1*A*) responsive to estradiol-activated XVE (27). The promoter sequence of *FER* (*pFER*) gives endogenous expression to *FER-pafA* (described in Fig. 1A). *B*, Schematic Diagram of PUP-IT for FER-interaction tagging in Arabidopsis seedlings. Five-day-old FER PUP-IT transgenic seedlings were treated for 2 days with estradiol to induce *FLAG-pupE* or with diluted DMSO alone to leave expression uninduced. PM-localized FER-PafA ligated FLAG-PupE to prey proteins. FLAG-tagged prey proteins were immunoprecipitated on anti-FLAG antibody-beads. Mass spectrometric analysis was used to identify peptides from enriched prey proteins. *C*, the overlap of three sets of candidate proteins from three replicated experiments is depicted in a Venn diagram with numbers and percentages of proteins. *D*, Volcano plot of the 1964 proteins (*red and grey*) identified by LC–MS/MS in the seedling. The −log_10_ (*p*-value) is plotted against log_2_ (fold change: abundant proteins in the experiment group/abundant proteins in the control group). The non-axial vertical lines denote ±1.0-fold change while the non-axial horizontal line denotes *p* = 0.05. The *red dots* represent 182 proteins with significant differences between the experimental group and the control (*p*-value <0.05 and fold change >1).

Surveys of proteins in protoplasts would be limited to the proteins and processes that occur in leaf mesophyll cells. To more broadly survey proteins that possibly come into proximity to FER in other organs and cell types or at particular developmental stages, we generated transgenic lines that stably inherit *FER-pafA* and *FLAG-pupE*. To recapitulate a native transcriptional expression and avoid ectopic expression of FER that might adversely affect plant growth, we directed FER-PafA expression using the endogenous promoter sequence of *FER* (Fig. 2*A*) (21). To limit background, FLAG-PupE expression was under the control of an established estrogen-inducible expression system using an additional gene constitutively expressing the estrogen receptor-based transactivator (XVE) (28). The three genes (*XVE*, *FLAG-pupE* and *FER-pafA*) were assembled into the T-DNA region of a single binary vector so that Agrobacterium-mediated transformation would introduce the three genes into the chromosome as a single locus in transgenic lines (Fig. 2*A*).

We profiled FER-proximal proteins in five-day-old seedlings harboring the *XVE*, *FLAG-pupE*, and *FER-pafA* transgenes. Expression of *FLAG-pupE* was induced by adding estradiol to liquid cultures (Supplemental Fig. S4*B*). Two days after treatment seedlings were harvested, and pupylated FLAG-tagged proteins were immunoprecipitated and enriched from lysates of harvested seedlings using immobilized anti-FLAG antibodies (Fig. 2*B*). Analysis of peptide fragments of proteins in three replicated sets led to the identification of 2963 Arabidopsis proteins in total (Fig. 2*C* and Supplemental Table S7). Among total proteins, 329 were present in all three sets and 669 were in just two sets. The numbers and percentages of identified proteins in sets from replicated experiments and the overlap among sets is shown in Figure 2C.

We similarly profiled proteins from 5-day-old transgenic seedlings treated for 2 days with the diluted solvent (DMSO) used to dissolve estradiol. In total 5991 Arabidopsis proteins could be identified in three sets from replicated experiments (Supplemental Table S8). Because expression of *FLAG-pupE* would remain uninduced in the absence of added estradiol, we expected proteins obtained from our enrichment scheme would largely include contaminants lacking a FLAG-epitope tag that were nevertheless non-specifically isolated from lysates. Interestingly, among proteins specifically enriched after induction of *FLAG-pupE* were proteins previously characterized as FER-interacting, namely GUANINE EXCHANGE FACTOR 4 (GEF4, At2g45890), BRI1-ASSOCIATED RECEPTOR KINASE 1 (BAK1, At4g33430), Armadillo (ARM)-repeat protein At4g16490, PLANT U-BOX 9 (PUB9, At3g07360), HETEROGLYCAN GLUCOSIDASE 1 (HGL1, At3g23640) and MYC2 (At1g32640) (36, 37, 38, 39). Among the proteins which analyzed in volcano plot, 182 were determined to be significantly enriched in samples in which proteins were pupylated by FER-PafA (*p*-value <0.05 and a fold change >1 cutoff, Fig. 2*D*, and Supplemental Tables S9 and S10).

### Surveying FER-Interacting Proteins in Flowers

**Figure 3:**
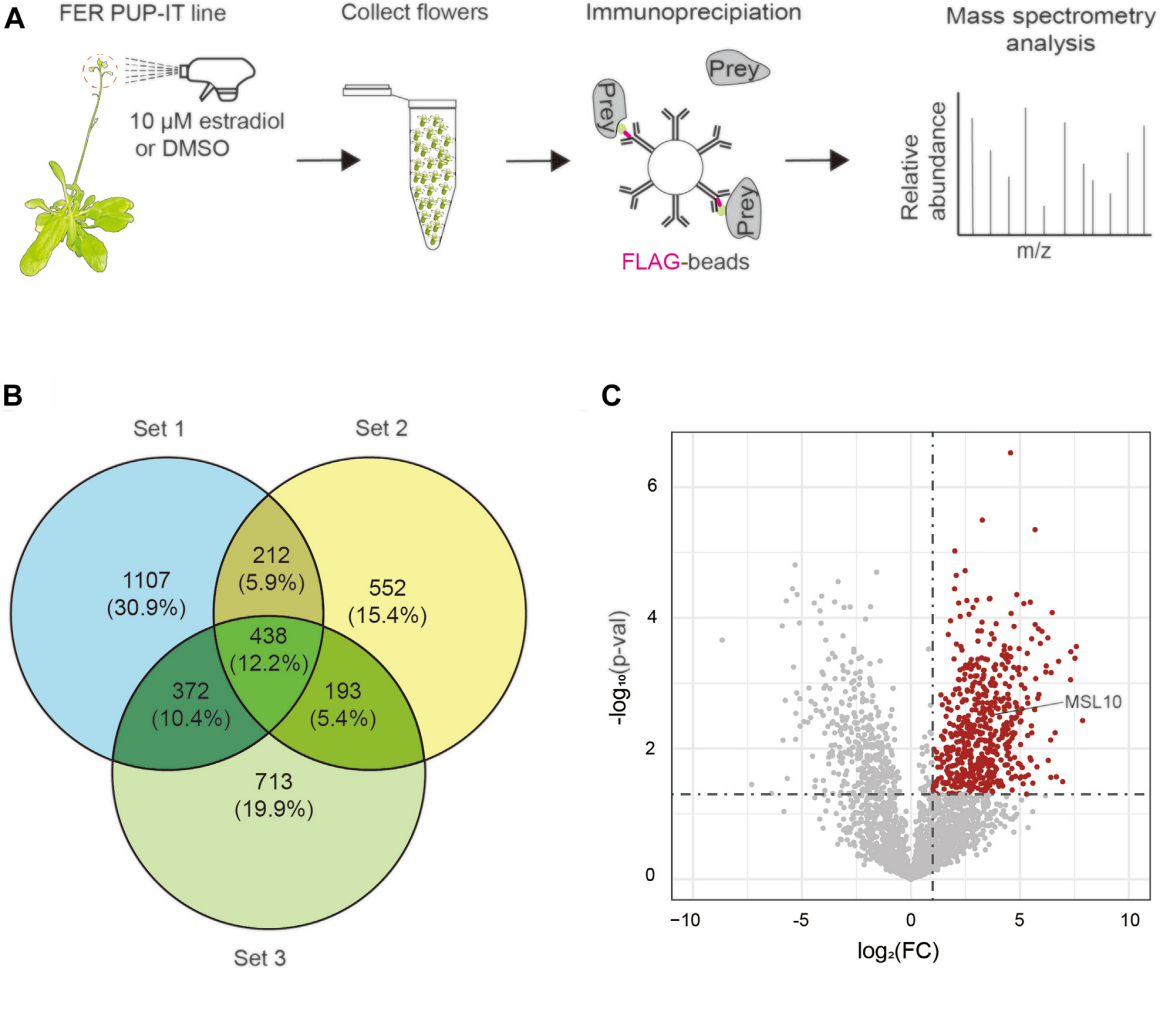
**Identifying FER-proximal proteins by PUP-IT analyses in Arabidopsis flowers.***A*, Schematic diagram of PUP-IT for FER-interaction tagging in Arabidopsis flowers. Inflorescences of 35-day-old transgenic FER PUP-IT plants were sprayed twice daily for 3 days with estradiol to induce expression of *FLAG-pup* or with DMSO to leave *FLAG-pupE* uninduced. Flowers were collected, and pupylated prey proteins were enriched with immunoprecipitation on anti-FLAG antibody-beads. Mass spectrometric analysis of peptides of enriched proteins identified candidate FER-interacting proteins. *B*, the overlap of three sets of candidate proteins from three replicated experiments is depicted in a Venn diagram with numbers and percentages of proteins. *C*, Volcano plot of the 2572 (*red and grey*) proteins identified by LC–MS/MS in flower. The −log_10_ (*p*-value) is plotted against the log_2_ (fold change: abundant proteins in the experiment group/abundant proteins in the control group). The non-axial vertical lines denote ±1.0-fold change while the non-axial horizontal line denotes *p* = 0.05. The *red dots* represent 565 proteins with significant differences between the experimental group and the control (*p*-value <0.05 and fold change >1). The *grey dots* represent 2007 proteins showing no significant difference in flowers.

As *FER* plays crucial role in fertilization, we performed surveys of FER-interacting proteins that focused on flowers where fertilization occurs (Fig. 2*A*). To make the survey include all stages of fertilization, we treated young indeterminant inflorescences with estradiol to induce strong expression of *FLAG-pupE* in the same transgenic line used for surveying FER-proximal proteins in seedlings (Supplemental Fig. S4*C*). Flowers were harvested 3 days after treatment, and FLAG-epitope tagged proteins were enriched with immobilized anti-FLAG antibody on magnetic beads (Fig. 3*A*). In three replicated samples collected after induction of *FLAG-pupE*, peptide fragments of enriched proteins were assigned to 3621 Arabidopsis proteins (see Fig. 3*B* and Supplemental Table S11). Among the identified proteins from flowers, 438 were found in all three replications, and 777 were in at least two replications. The numbers and percentages of proteins assigned to each replicate, as well as the overlap among replicates are illustrated in Figure 3*B*.

In three replications, flowers were treated with diluted solvent (DMSO) alone so that *FLAG-pupE* remained uninduced (Supplemental Table S12). From the enrichment of lysates of flowers harvested 3 days later, peptides could be assigned to a total of 5165 proteins in the reference set of Arabidopsis proteins; and, among replicates 492 proteins were identified in three samples (Supplemental Table S12). Interestingly, proteins corresponding to genes associated with the function of FER, namely ANJEA (ANJ, At5g59700), HERCULES RECEPTOR KINASE 1 (HERK1, At3g46290), ROP GUANINE NUCLEOTIDE EXCHANGE FACTOR 2 (ROPGEF2, At1g01700), TARGET OF RAPAMYCIN (TOR, At1g50030), and C2-DOMAIN ABA-RELATED 10 (CAR10, At2g01540), were specifically identified among proteins enriched in flowers after induction of *FLAG-pupE* (22, 40, 41, 42). We generated a volcano plot to emphasize noteworthy proteins pupylated by PafA under conditions with a *p*-value <0.05 and a fold change >1 cutoff (Fig. 3*C*, and Supplemental Tables S13 and S14). Among the proteins that were analysed, 565 were identified as significantly different between the experimental group and the control (see volcano plots in Figs. 1*D*, 2*D* and 3*C*). No proteins were common to the three sources of tissue (protoplasts, seedlings and flowers). 10, 14 and 22 proteins were significantly enriched in samples from two sources, protoplasts/seedlings, flowers/seedlings and flowers/protoplasts, respectively (Supplemental Fig. S5 and Supplemental Table S15).

### Validation of Proximity Labeling of Selected Candidate Proteins

**Figure 4:**
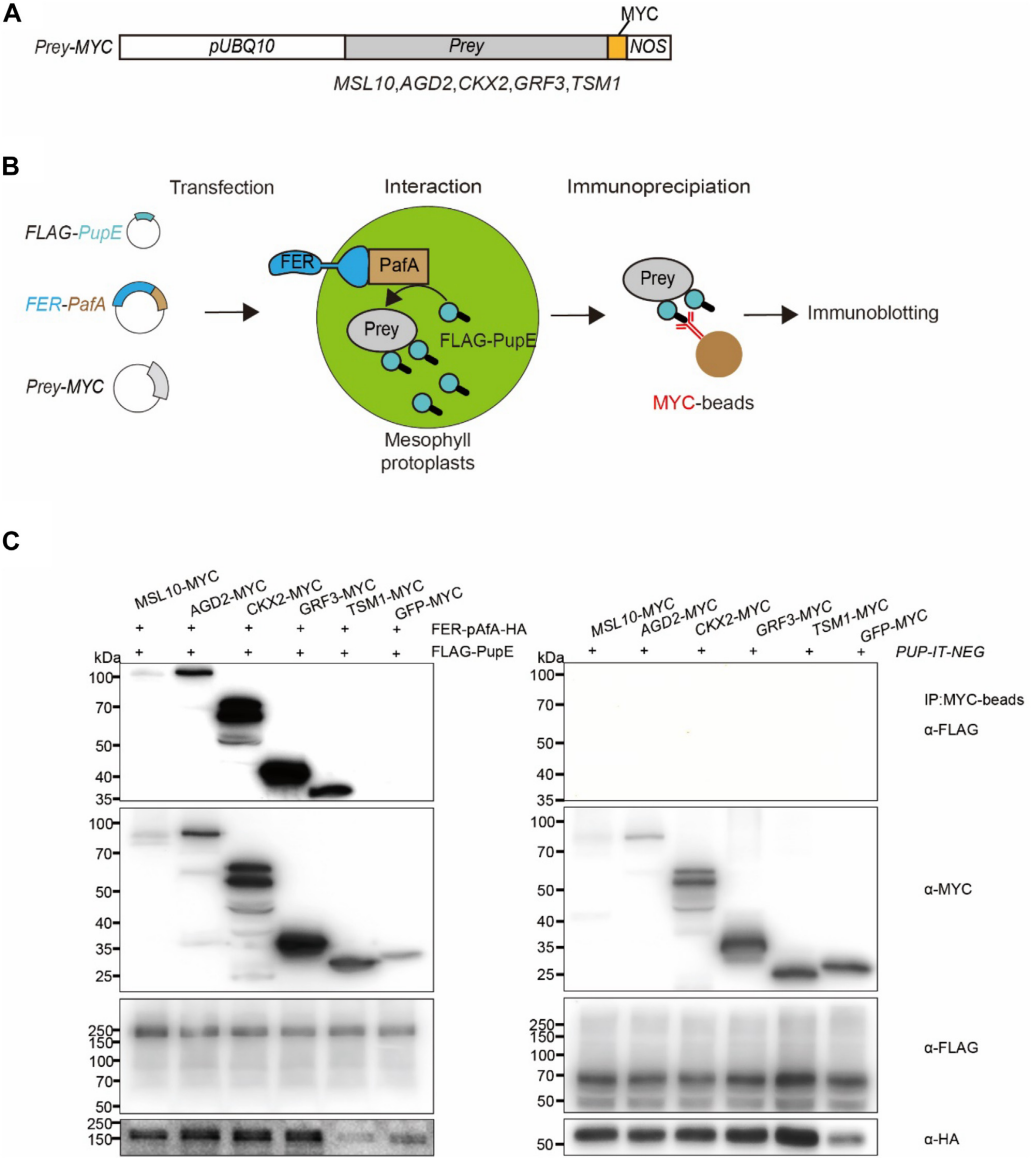
**Validation of candidate FER-Interacting Proteins in Arabidopsis protoplasts.***A*, *Prey-MYC* constructs for transient expression of candidate prey proteins in protoplasts. The Arabidopsis *UBQ10* promoter (*pUBQ10*) and *NOS* sequences respectively flank start and stop codons. Constructs include coding sequences of *MSL10*, *AGD2*, *CKX2*, *GFR3*, *TSM1*, or *GFP* (negative control) and two copies of the MYC epitope. *B*, Schematic diagram of PUP-IT for validating FER-interacting candidate prey proteins in mesophyll protoplasts. *Prey-MYC* constructs were transiently co-expressed with *FLAG-pup* and *FER-pafA* overnight in transfected protoplasts, FLAG-Pup tags on Prey-MYC captured interactions with FER-pafA. Prey-MYC proteins were immunoprecipitated (IP) with anti-MYC antibodies immobilized on agarose beads (MYC-beads), separated in SDS-PAGE and immunoblotted with antibodies to FLAG (α-FLAG), HA (α-HA) and MYC (α-cMYC) epitopes. *C*, validating FER-interacting candidate prey proteins. α-cMYC detected bands with gel mobilities unique to expressed Prey-MYC proteins. MSL10 (83 kDa), AGD2 (88 kDa), CKX2 (56 kDa), GRF3 (33 kDa), TSM1 (26 kDa) and GFP (28 kDa); and, α-HA detected a band with gel mobility expected for FER-PafA-HA (155 kDa) and PafA-HA (58 kDa). On immunoblots of IP on MYC-beads, α-FLAG detected bands with gel mobility unique to expressed Prey-MYC proteins (*right*) and failed to detect bands for the negative control (*left*) (from expression of *GFP-MYC* or *PUP-IT NEG*). kDa, kilodaltons.

In transfected protoplasts, we confirmed the proximity labeling of protein products of five novel candidate genes (Fig. 4*A*). Among genes encoding proteins identified as potential targets of FER-PafA, we selected *MECHANOSENSITIVE CHANNEL OF SMALL CONDUCTANCE-LIKE 1* (*MSL10*, At5g12080), *ARF-GAP DOMAIN 2* (*AGD2*, At1g60860), *CYTOKININ OXIDASE 2* (*CKX2*, At2g19500), *TAPETUM-SPECIFIC METHYLTRANSFERASE 1* (*TSM1*, At1g67990), and *GROWTH-REGULATING FACTOR 3* (*GRF3*, At2g36400) for validation. Candidate genes that encoded fusions of the MYC epitope to the carboxy-termini of the coding sequences of putative prey proteins (Prey-MYC) were transiently expressed in protoplasts. Individual Prey-MYC constructs were co-expressed overnight in transfected protoplasts with either *FER-pafA* and *FLAG-pupE* or *pafA* and *FLAG-pupE* (serving as a negative control). Proteins were immunoprecipitated from cell lysates with anti-MYC antibodies first and then analyzed by immunoblot analyses to detect the proteins carrying both MYC- and FLAG-epitopes (Fig. 4*B*). The relative position of the bands detected with both anti-MYC, and anti-FLAG antibodies were unique to the proteins derived from transfections of different *Prey-MYC* constructs.

## Discussion

Using the proximity labeling of FER-PafA, we surveyed proteins in Arabidopsis mesophyll protoplasts, seedlings, and flowers that could potentially interact with FER. We took advantage of transient gene expression in protoplasts to expedite the testing of a new construct expressing FER-PafA and to verify the proximity labeling of five novel candidate FER-interacting proteins. Stable expression of FER-PafA in the transgenic line *FER-PUP-IT* was used to survey FER-proximal proteins in whole seedlings and flowers. Transgenic plants could be further used to identify FER-proximal proteins in other organs, tissues, and developmental stages or when plants are grown with appropriate nutrient, abiotic, or biotic stimuli.

For PUP-IT analyses in protoplasts, we also generated LC-MS/MS results from the expression of PafA alone without fusion to FER. Our intention was to identify prey proteins in proximity to FER but not false-positive interactions with PafA. To account of such false positives, we compared results from transient expression of PafA and FER-PafA. This comparison is imperfect because mature FER-PafA should localize to the plasma membrane while PafA is cytosolic. Driven by the constitutive promoter, HBT, proteins can be detected in expression 2 h after transfection in the protoplast assay system (27). In our assay, we aimed to maximize PupE labeling. However, this could potentially increase false positive or false negative results. Increasing the number of protoplasts and reducing the incubation time for protein expression may reduce false background results. The distinct cellular environments could contribute to differences in the proximity labeling of PafA and FER-PafA. Nevertheless, we showed the value of accounting for false-positives candidates when using PUP-IT analyses for surveys.

Pupylation presents some features that distinguish it from biotinylation in proximity labeling. PupE must be heterologously expressed in plant and animal cells undergoing pupylation, and the choice of transcriptional promoters for expressing PupE could be used to promote proximity labeling in specific organs, tissues, cell types, or at certain developmental stages. We incorporated an established estradiol-inducible gene expression system to express FLAG-PupE, which made temporal control of pupylation possible. In addition, by performing PUP-IT analyses in the absence of estradiol-induction of FLAG-PupE, it was possible to account for false-positive results from our enrichment scheme for FLAG-tagged proteins. For proximity labeling in subcellular compartments, signal sequences for subcellular localization would need to be fused to the amino terminus of PupE. For enrichment and detection of pupylation, sequences encoding epitopes or affinity tags are fused to PupE. For instance, in the original report of the PUP-IT method, a sequence that is naturally biotinylated was fused to PupE. We chose to fuse FLAG epitopes as well as a histidine divalent metal-affinity tag to the amino terminus of PupE for further reduction of false positive background by dual immunoprecipitation steps in future applications.

The overlap of proteins identified in separate sets (or replicates, as shown in Figs. 1*C*, 2*C*, and 3*B*) was low. We noticed that most proteins found in multiple sets (greater than 90%) were identified with low numbers of defined peptides (two or fewer). Counterintuitively, this suggests that proximity tagging was more reproducible between FER and prey proteins with weaker or more transient interactions or that reproducible interactions were more often made with less abundant prey proteins. In fact, the five novel candidate proteins that we have confirmed were identified with low numbers of defined peptides. Moreover, among the 14 previously characterized FER-interacting proteins identified in our surveys, 13 were found with low numbers of defined peptides.

The above observation suggests possible improvements for future surveys using PUP-IT. For instance, the number of sets or replicates could be increased to avoid missing reproducible but poorly detected candidate proteins, which might be overlooked in a single experimental set. Also, it might be possible to increase the number of defined peptides by changing the settings of chromatography and mass spectrometry, such as increasing the separation of peptides to prolong the gradient running time and gain more peptide information, which should improve the definition of associated proteins.

In the cases of controls without induction of FLAG-pupE, identified proteins in the sets of controls were expected to be fewer than, and largely a subset of, the proteins in the experimental sets. However, numerous proteins were identified in the sets of controls that were absent in the experimental sets. We suspect the appearance of control-specific proteins was due to the reduced abundance of proteins without epitope tags, which were obscured in experimental sets by the more abundant epitope-tagged proteins. We include a list of these non-epitope-specific proteins that were evident in control sets.

Identified proteins from each source of tissue were analyzed for significant differences in enrichment in the experimental and control sets (as visualized in volcano plots). No proteins were found in all three sources, which might reveal differences in signaling pathways and protein interaction networks of FER among different tissues. However, some proteins were found in all combinations of two of three sources, which also suggests that these sources share some common and prevalent components in FER signaling. These latter proteins deserve further investigation.

Among candidate proteins from protoplasts, seedlings, and flowers, 14 were previously reported to be FER-interacting proteins (Supplemental Table S16). Among membrane-localized candidates, the RLK HERK1, which was detected in surveys of flowers, interacts with the related CrRLK1L kinases FER and THESEUS1 and coreceptor LORELEI. Together, these receptors form complexes to control female determinants of fertilization by pollen (40). Detected in surveys of seedlings, the RLK BAK1 is a coreceptor of pattern-recognition receptors such as FLAGELLIN-SENSITIVE 2 (FLS2), and FER stabilizes the formation of the receptor complex of BAK1 and FLS2 in the presence of the flagellin peptide ligand Flg22 (38). Detected in surveys of seedlings and flowers, AHA2 is an integral membrane proton-ATPase pump and is phosphorylated by FER (34).

Not all previously reported FER-interacting proteins were detected in our surveys. Possibly interactions with such proteins are conditional, and the conditions were not met during our surveys. For instance, in response to a high Carbon (C)/Nitrogen (N) ratio, FER phosphorylates the E3 ubiquitin ligase ATL6, which modulates plant growth. ATL6 was not a candidate protein or even identified in individual replicates (43). However, the growth conditions in our study included a balanced C/N ratio. The detection of such an interaction may require choosing appropriate environmental conditions or stimuli when performing surveys.

Our surveys also identified novel FER-interacting candidates involved in processes in which FER has characterized roles. FER regulates cell growth and responses to various hormones. In particular, brassinosteroid, abscisic acid, ethylene, auxin, and jasmonic acid have crosstalk with FER to regulate plant development as well as defense against bacteria and fungi (18). Cytokinin oxidase/dehydrogenase CKX2, which inactivates cytokinin and thus controls cytokinin activity, was identified in surveys of protoplasts, seedlings, and flowers (44). The plant hormone cytokinin is a crucial modulator of plant growth and development (45). The reproducible detection of the interaction of CKX2 and FER implies a possible role for FER in cytokinin homeostasis. A novel pathway regulating the activity of cytokinin might be revealed in future work to characterize the FER-CKX2 interaction.

The mechanosensitive (MS) ion channel opens and conducts ions in response to different stimuli. MSL10 is a member of the MS ion channel family in Arabidopsis and promotes a cytosolic Ca^2+^ transient accumulation of reactive oxygen species when activated by cell swelling (46). A defective mechanoperception is observed in *fer*, and FER is proposed to act as a mechano-sensor that senses cell wall tension (21). Interestingly, MSL10 was identified as a putative FER-interacting protein, which suggests a possible link between the channel activity of MSL10 and the signal transduction of FER. The observed FER-MSL10 interaction suggests hypotheses about the integration of signals in mechanoperception, which could now be pursued.

In protoplasts expressing *FER-pafA* and *FLAG-pupE*, we tested the pupylation of five candidate proteins MSL10, CKX2, AGD2, TSM1, and GRF3. AGD2, which was detected in surveys of protoplasts and seedlings, encodes an ADP ribosylation factor GTPase activating protein and plays a crucial role in regulating vesicle trafficking of auxin efflux regulators (47). TSM1 encodes a tapetum-specific O-methyltransferase and is important in stamen/pollen development (48). GRF3, which was identified in PUP-IT surveys of flowers, is a 14-3-3 protein, generally binding to phosphorylated proteins, and can contribute to the regulation of various signaling cascades. Specific pupylation of these five proteins (but not the control protein GFP) supported our expectation that detected proteins in our initial surveys for FER-interacting proteins were in fact enriched for FLAG-tagged pupylated proteins (Fig. 4B).

## Data Availability

The datasets analysed in this article are available online. The mass spectrometry proteomics data have been deposited to the ProteomeXchange Consortium *via* the PRIDE partner repository with the dataset identifier PXD045546 and 10.6019/PXD045546 and PXD052510 (Username: reviewer_pxd045546@ebi.ac.uk Password: zdOn9NsH and Username: reviewer_pxd052510@ebi.ac.uk Password: eDCxnBYRY8F0) (49). Project Name: Profiling FERONIA interaction proteins by Pupylation-based interacting tagging in Arabidopsis. Project accession: PXD045546 and PXD052510. Project DOI: 10.6019/. The data access connection in ProteomeXchange is http://proteomecentral.proteomexchange.org/cgi/GetDataset?ID=PXD045546.

## Supplemental data

This article contains supplemental data.

## Conflict of interest

The authors declare that they have no conflict of interest with the contents of this article.

## Supplementary Data

Supplemental Table S1 The R script was used for performed for volcano plot and PCA analysis.

Supplemental Table S2 Oligonucleotide sequences of PCR primers used for cloning.

Supplemental Table S3 FER-proximal proteins in protoplast identified by MS.

Supplemental Table S4 Proteins from protoplasts identified by MS in negative control samples.

Supplemental Table S5 Statistical analysis of quantities of proteins identified in experimental and control samples from protoplasts.

Supplemental Table S6 Individual proteins in samples from protoplasts included in volcano plot.

Supplemental Table S7 FER-proximal proteins in seedlings identified by MS.

Supplemental Table S8 Proteins from samples of seedlings identified by MS in negative control.

Supplemental Table S9 Statistical analysis of quantities of proteins identified in experimental and control samples from seedlings.

Supplemental Table S10 Individual proteins in samples from seedlings included in volcano plot.

Supplemental Table S11 FER-proximal proteins in flower identified by MS.

Supplemental Table S12 Proteins from samples of flowers identified by MS in negative control samples.

Supplemental Table S13 Statistical analysis of quantities of proteins identified in experimental and control samples from flowers.

Supplemental Table S14 Individual proteins in samples from flowers included in volcano plot.

Supplemental Table S15 Individual candidate FER-interacting protein from protoplasts, seedlings and flowers.

Supplemental Table S16 Proteins previously characterized as FER-interacting.
