# Supplementary material for: Pupylation-Based Proximity-Tagging of FERONIA-Interacting Proteins in Arabidopsis
Source: Mol Cell Proteomics. 2024 Aug 13;23(11):100828. doi: 10.1016/j.mcpro.2024.100828 (PMC11532908; doi:10.1016/j.mcpro.2024.100828)
Supplement: Supplemental Figure S1 [file mmc17.pdf]

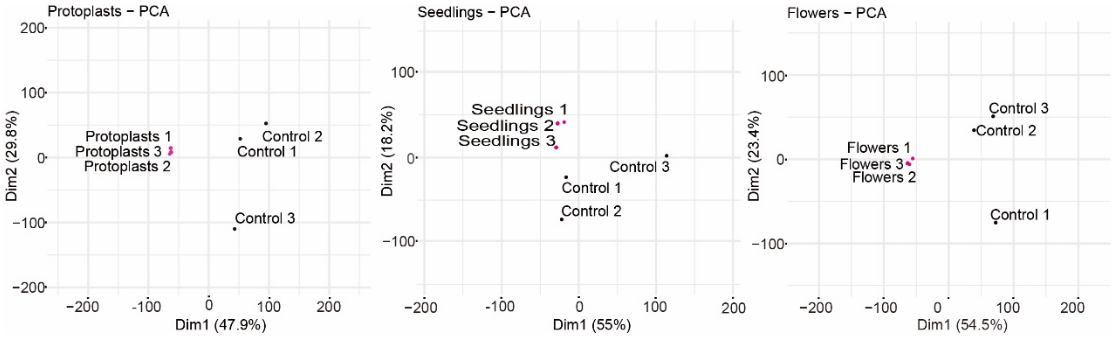

Fig.S1. PCA analysis of MS data from protoplasts, seedlings and flowers

The PCA analysis of proteins identified in experimental group and control group.
