# Supplementary material for: Pupylation-Based Proximity-Tagging of FERONIA-Interacting Proteins in Arabidopsis
Source: Mol Cell Proteomics. 2024 Aug 13;23(11):100828. doi: 10.1016/j.mcpro.2024.100828 (PMC11532908; doi:10.1016/j.mcpro.2024.100828)
Supplement: Supplemental Figure S2 [file mmc18.pdf]

6XHis-3XFLAG-PupE

ATGCGTGGATCTCATCATCATCATCATCATTCTCAAGATCCTGACTACAAAGACCATGACG-
GTGATTATAAAGATCATGACATCGACTACAAGGATGACGATGACAAGATGAACGCAAAGCAAACCCAAA
TTATGGGTGGCGGCGGACGTGACGAGGACAACGCCGAGGATTCCGCACAGGCATCTGGACAGGTTC
AGATCAACACCGAAGGTGTGGACAGCTTGCTGGATGAAATCGACGGACTGTTGGAAAACAACGCCGA
GGAATTCGTTCGTTCCTATGTACAAAAGGGTGGCGAATGA

PafA-GS-linker

ATGAGTACCGTGGAATCCGCATTGACCCGCAGGATCATGGGCATTGAAACGGAGTATGG-
CCTCACCTTTGTTGATGGTGATTCCAAAAAGCTTCGCCCAGATGAGATAGCTCGAAGGATGTTTCGTC
CCATCGTGGAGAAATATTCCAGCTCTAATATCTTCATACCCAATGGTTCCCGGTTGTATCTTGATGTGG
GTTCCCACCCGGAGTACGCCACCGCCGAGTGTGATAATTTGACCCAGCTGATCAATTTTGAAAAAGCT
GGCGATGTTATTGCAGATCGCATGGCTGTAGATGCCGAAGAGTCGCTGGCGAAAGAAGACATTGCTG
GGCAGGTGTACCTGTTTAAAAACAATGTCGATTCCGTGGGCAATTCTTATGGCTGCCACGAAAACTAC
CTTGTGGGTCGCTCCATGCCGTTGAAGGCGTTGGGTAAAAGGCTGATGCCGTTTCTGATTACCCGCC
AGCTCATCTGCGGCGCCGGCAGGATCCATCACCCCAATCCTTTGGATAAAGGCGAATCCTTCCCCTT
GGGCTACTGCATATCCCAGCGCTCTGACCACGTGTGGGAGGGCGTATCAAGTGCCACCACTAGATCA
CGCCCCATTATCAACACCCGTGATGAGCCACATGCGGATTCCCATTCTTACCGCAGGCTGCACGTGAT
TGTGGGTGATGCCAACATGGCAGAGCCCAGCATCGCGTTGAAGGTCGGCTCCACGTTGCTGGTTCT
GGAAATGATTGAGGCAGATTTCGGTTTGCCCAGCTTAGAGCTTGCCAATGATATTGCCTCAATTAGGG
AAATCTCCCGCGATGCAACAGGATCCACACTGTTGTCCCTGAAAGATGGCACCACCATGACTGCCTT
GCAGATCCAGCAGGTGGTCTTTGAGCATGCCTCGAAGTGGTTGGAGCAGCGCCCCGAACCAGAATT
TTCTGGCACCTCCAACACAGAGATGGCCCGCGTGCTGGATCTGTGGGGTCGCATGTTGAAAGCGATT
GAGTCCGGTGATTTCAGCGAAGTGGATACAGAAATTGACTGGGTGATCAAAAAGAAGCTCATTGATCG
TTTCATTCAGCGCGGCAACCTTGGGTTGGATGATCCAAAACTTGCCCAAGTGGACTTGACTTATCACG
ATATTAGGCCAGGTAGAGGCCTATTTAGCGTGCTGCAAAGCCGCGGCATGATCAAACGGTGGACTACT
GATGAGGCGATTTTAGCTGCGGTGGATACCGCTCCTGATACAACACGTGCTCATTTGCGCGGGCGAA
TCCTTAAAGCGGCGGATACTCTGGGAGTACCTGTGACTGTCGATTGGATGCGTCACAAGGTCAACCG
ACCGGAGCCACAATCGGTGGAATTGGGGGATCCTTTTTCCGCAGTGAATTCCGAGGTTGATCAGTTG
ATTGAGTATATGACCGTTCATGCGGAGAGCTATCGCAGTGGAGGAGGATCTGGAGGAGGATCTGGAG
GAGGATCTGGAGGAGGATCT

Fig. S2. Sequence of 6XHis-3XFLAG-PupE and PafA-GS-linker.
6xHis-3xFLAG is fused to the N terminus of full-length Pup (pink). The 48bp GS-linker (black) is fused to the C terminus of PafA (blue).
