# Supplementary material for: Pupylation-Based Proximity-Tagging of FERONIA-Interacting Proteins in Arabidopsis
Source: Mol Cell Proteomics. 2024 Aug 13;23(11):100828. doi: 10.1016/j.mcpro.2024.100828 (PMC11532908; doi:10.1016/j.mcpro.2024.100828)
Supplement: Supplemental Figure S3 [file mmc19.pdf]

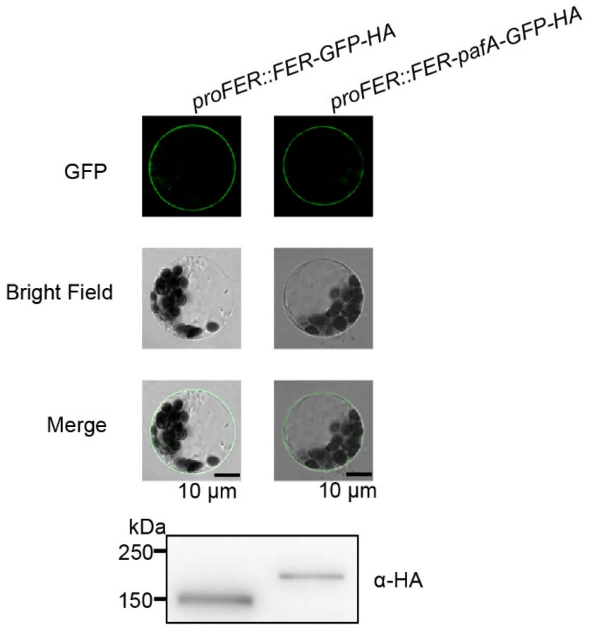


**Fig.S3. Expression and subcellular location of FER-GFP-HA and FER-pafA-GFP-HA.** Fluorescent signal accumulated at the plasma membrane of Arabidopsis mesophyll protoplast expressing *FER-GFP-HA* or *FER-pafA-GFP-HA* using the native promoter sequence of *FER*. α-HA antibody detected bands with gel mobility consistent with the expected molecular weight of FER-GFP-HA (126.7 kDa) and FER-pafA-GFP-HA (181.8 kDa).
