# Supplementary material for: Pupylation-Based Proximity-Tagging of FERONIA-Interacting Proteins in Arabidopsis
Source: Mol Cell Proteomics. 2024 Aug 13;23(11):100828. doi: 10.1016/j.mcpro.2024.100828 (PMC11532908; doi:10.1016/j.mcpro.2024.100828)
Supplement: Supplemental Figure S4 [file mmc20.pdf]

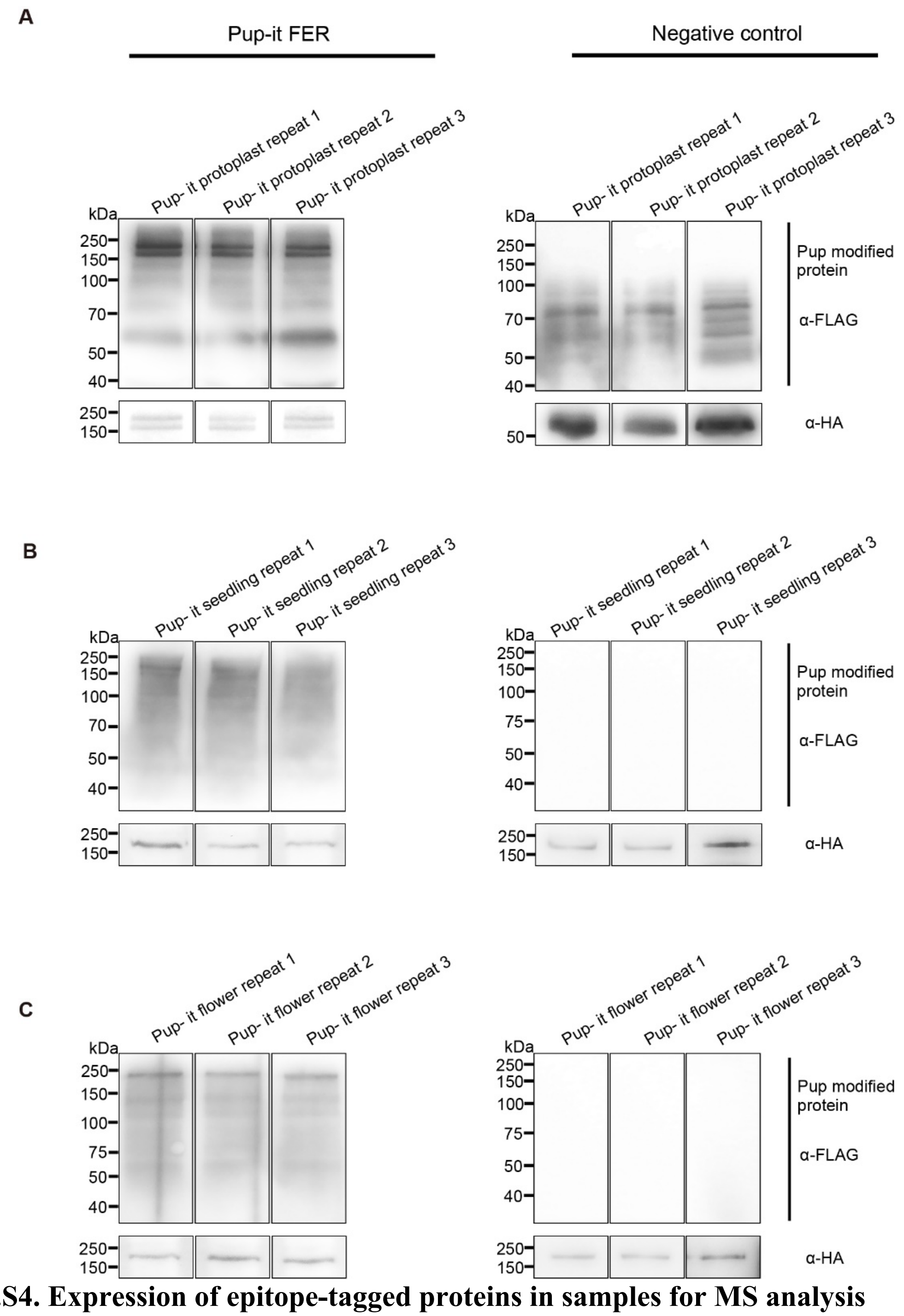

Fig.S4. Expression of epitope-tagged proteins in samples for MS analysis

Chemiluminescence of immunoblots of samples of three repeats of Pup-it FER (left side) and three repeats of the negative control (right side) from protoplasts (A), seedlings (B) and flowers (C). On Western blots of total protein in lysates, α-FLAG antibody detected a smear of bands resulting from Pup modification of proteins proximal to FER-pafA-HA or pafA-HA. Anti-HA antibody detected bands with the expected mobility of FER-pafA-HA (155 kDa) and pafA-HA (55 kDa).
