# Supplementary material for: Pupylation-Based Proximity-Tagging of FERONIA-Interacting Proteins in Arabidopsis
Source: Mol Cell Proteomics. 2024 Aug 13;23(11):100828. doi: 10.1016/j.mcpro.2024.100828 (PMC11532908; doi:10.1016/j.mcpro.2024.100828)
Supplement: Supplemental Figure S5 [file mmc21.pdf]

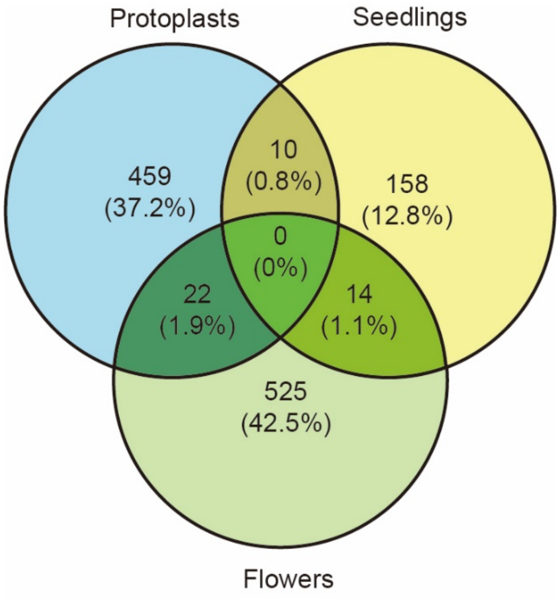

Fig.S5. Venn diagram of the overlap of three experimental groups.

The overlap of three experimental groups (protoplasts, seedlings and flowers) of candidate proteins from three replicated experiments is depicted in a Venn diagram with numbers and percentages of proteins.
